# No evidence for association between polygenic risk of multiple sclerosis and MRI phenotypes in ~30,000 healthy adult UK Biobank participants

**DOI:** 10.1177/13524585221075744

**Published:** 2022-03-16

**Authors:** Benjamin Meir Jacobs, Cameron Watson, Charles Marshall, Alastair Noyce, Ruth Dobson

**Affiliations:** 1 Preventive Neurology Unit, Wolfson Institute of Population Health, Queen Mary University of London, London, UK; 2 Department of Neurology, Royal London Hospital, London, UK

De Mol et al.^1^ recently published an analysis of the correlation between genetic risk for multiple sclerosis (MS) and magnetic resonance imaging (MRI) metrics of white matter tract integrity in healthy children. They used an MS polygenic risk score (PRS) derived from the International Multiple Sclerosis Genetics Consortium (IMSGC) genome-wide association study (GWAS)^2^ summary statistics. In a cohort of 1087 healthy, unrelated children of European ancestry, the PRS was associated with a number of MRI ‘molehills’ – localised increases in fractional anisotropy (FA) indicative of focal white matter tract alterations. This extends the previously reported correlation between higher MS genetic risk and global FA in the same cohort.^3^

It is unclear whether these observations extend to healthy adults. Ikram et al.^4^ reported nominal associations between a 110-variant MS PRS and various volumetric and tractographic MRI outcomes in 4710 healthy adults; however, none of these associations survived multiple testing. An analysis of an early release of UK Biobank (UKB) MRI data (*n* = 8353) found no association between an MS PRS and white matter hyperintensity volume, global FA, or mean diffusivity.^5^

We sought to replicate the results of De Mol et al. in the latest release of UKB data, which contain MRI data for ~50,000 individuals. For MRI outcomes, we used imaging-derived phenotypes (IDP) produced by a standardised pipeline on behalf of UKB.^6^ People with cerebral small vessel disease, Alzheimer’s disease, and Parkinson’s disease (defined by the ‘source of report’ fields in UKB, which combine self-report, Hospital Episode Statistics, primary care codes, and other sources) were excluded to avoid confounding. A variety of MS polygenic risk scores were generated using the clumping-and-thresholding approach (full details and code available at https://github.com/benjacobs123456/PRS_UKB_MRI, methods similar to those previously described^7^). Scores were calculated by both including the major histocompatibility (MHC) region (using the HLA-DRB1*15:01 risk allele to capture the risk conferred by variation at this locus^8^) and excluding this region. In all, 130 distinct PRSs (65 non-MHC, 65 with MHC) were generated by varying the clumping *p*-value threshold (using 13 thresholds from 5 × 10^−8^ to 1) and the clumping *R*^2^ threshold (using five thresholds from 0.1 to 0.8). We divided the cohort into a training set comprising all unrelated individuals of European ancestry with no imaging data available (*n*_control_ = 346,547; *n*_ms_ = 1978) and a test set with imaging data available (*n*_control_ = 30,040; *n*_ms_ = 124). We identified the optimal PRS (explaining the maximal liability to MS as measured by Nagelkerke’s pseudo-*R*^2^ metric) with and without the MHC region included using the training set. We then applied these MHC-containing PRS and non-MHC PRS to the test set in order to investigate their association with MRI findings.

Within the testing set, both the MHC PRS and non-MHC PRS were strongly associated with MS susceptibility (non-MHC: *p* = 5.70 × 10^−6^, odds ratio (OR)_top-vs-bottom-decile_ = 3.64, 95% confidence interval (CI) = 1.57–8.44; MHC: *p* = 1.43 × 10^−7^, OR_top-vs-bottom-decile_ = 3.68, 95% CI = 1.68–8.07; logistic regression models adjusted for age, sex, and genetic principal components 1–4). These PRS explained 1.5% (MHC) and 1.3% (non-MHC) of MS susceptibility (Nagelkerke’s pseudo-*R*^2^ on the observed scale). As would be expected, individuals with MS had a higher total volume of T2 hyperintensities and demonstrated nearly global reductions in regional FA compared to healthy controls.

Neither the MHC-PRS (*p* = 0.34) nor the non-MHC-PRS (*p* = 0.49) was associated with T2 hyperintensity volume in healthy controls in linear regression models (*n* = 29,988, models adjusted as above plus total intracranial volume). Similarly, there was no association exceeding the multiple testing threshold (Bonferroni correction, alpha = 0.05, *n*_tests_ = 48) between either MHC-PRS or non-MHC PRS and regional FA in models adjusting for the same confounding covariates. We observed similar results over a range of *p*-value and clumping parameters.

Our results support earlier findings^5^ suggesting that, in healthy adults, MS polygenic risk does not correlate with white matter hyperintensity volume or regional FA. Although the PRS explains a small proportion of liability towards MS, the large sample size available here would enable us to detect a small effect of the PRS on MRI phenotypes (power > 99% for an *R*^2^ of 0.1%). The large sample size of UKB and a rigorous approach to selecting an optimal PRS score maximise the chance of observing such an effect. In summary, our results argue against the concept that healthy adult individuals at high genetic risk of MS have subclinical MRI evidence of the disease, in contrast to previous observations in children.
